# Numerical and experimental studies of stick–slip oscillations in drill-strings

**DOI:** 10.1007/s11071-017-3855-9

**Published:** 2017-10-31

**Authors:** Yang Liu, Joseph Páez Chávez, Rulston De Sa, Scott Walker

**Affiliations:** 1 College of Engineering Mathematics and Physical Sciences, University of Exeter, Rennes Drive, Exeter, EX4 4RN UK; 2 Faculty of Natural Sciences and Mathematics, Center for Applied Dynamical Systems and Computational Methods (CADSCOM), Escuela Superior Politécnica del Litoral, P.O. Box 09-01-5863, Guayaquil, Ecuador; 3 Department of Mathematics, Center for Dynamics, TU Dresden, 01062 Dresden, Germany; 4 School of Engineering, Robert Gordon University, Garthdee Road, Aberdeen, AB10 7GJ UK

**Keywords:** Drill-string, Stick–slip, Multistability, Non-smooth dynamical system, Numerical continuation

## Abstract

The cyclic nature of the stick–slip phenomenon may cause catastrophic failures in drill-strings or at the very least could lead to the wear of expensive equipment. Therefore, it is important to study the drilling parameters which can lead to stick–slip, in order to develop appropriate control methods for suppression. This paper studies the stick–slip oscillations encountered in drill-strings from both numerical and experimental points of view. The numerical part is carried out based on path-following methods for non-smooth dynamical systems, with a special focus on the multistability in drill-strings. Our analysis shows that, under a certain parameter window, the multistability can be used to steer the response of the drill-strings from a sticking equilibrium or stick–slip oscillation to an equilibrium with constant drill-bit rotation. In addition, a small-scale downhole drilling rig was implemented to conduct a parametric study of the stick–slip phenomenon. The parametric study involves the use of two flexible shafts with varying mechanical properties to observe the effects that would have on stick–slip during operation. Our experimental results demonstrate that varying some of the mechanical properties of the drill-string could in fact control the nature of stick–slip oscillations.

## Introduction

**Fig. 1:**
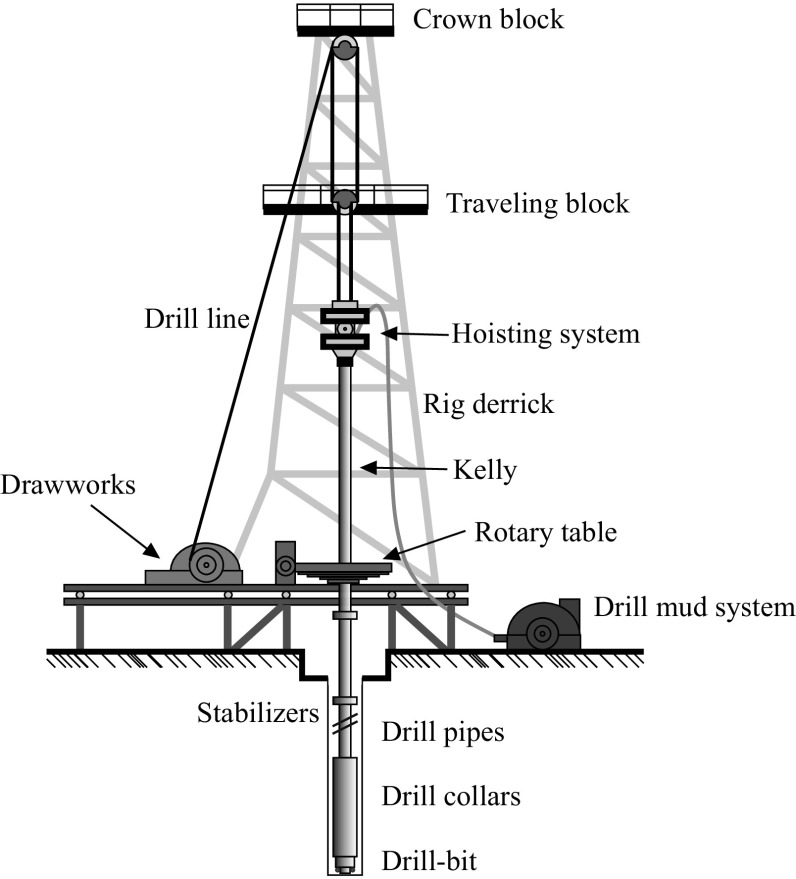
Schematic view of an oil well drill-string system (adopted from [2])

A drill-string is mainly comprised of a series of drill-pipes followed by a section known as the bottom-hole assembly (BHA), which consists of several moderately thicker drill collars that work in compression to supply the required weight on bit (WOB), and terminates with a drill-bit. Figure 1 presents a schematic view of a typical downhole drilling rig used in industry which includes the derrick, hoisting system, rotary table, drill-strings, drill-bit, and two drive systems to control the axial and rotational motions of the drill-strings. The first drive system employs an electrical motor coupled with a mechanical transmission box to provide torque to the rotary table at the surface. The rotary table is a large disk which functions as a kinetic energy storage unit used to sustain the desired rotational speed [1]. The rotary motion supplied by the rotary table is then transmitted through drill-strings to the drill-bit. Although the primary function of the drill-strings is to convey this rotary motion, they also provide the required axial force, namely the WOB, in order to facilitate the downhole drilling process. This axial force is normally controlled by the second drive system which incorporates the drill line and is powered by the drawworks.

**Fig. 2:**
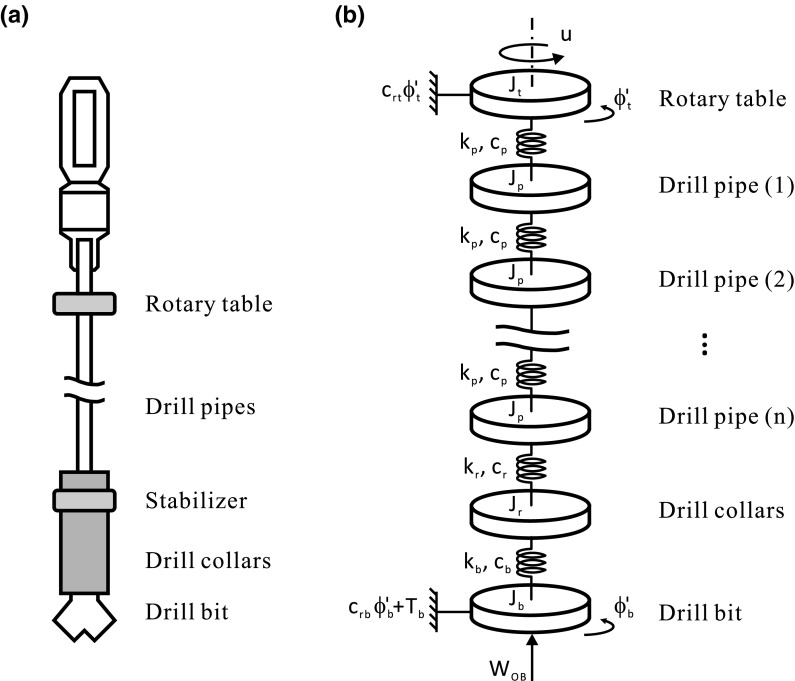
Schematics of **a** drill-string and **b** simplified drill-string system as considered in [3]

In practice, drill-strings are required to be driven at the desired constant speed maintaining the fastest rate of penetration into the rock formation. From a control system point of view, the drill-string structure is underactuated as it has one control input actuating on the rotary table from the surface and multi-degree-of-freedom downhole parts comprising the drill-pipes, drill collars, and drill-bit to be controlled. In the past few years, a number of mathematical models have been introduced to study the torsional Behaviour of the drill-strings during drilling operation; see [4] for a detailed review of the recent development. For example, Richard et al. [5] studied a simplified model to explore the root cause of stick–slip vibrations in drilling systems with drag bits, and Germay et al. [6] introduced a state-dependent delay at the bit–rock interface. A reduced-order model allowing for radial, bending, and torsion motions of a flexible drill-string and stick–slip interactions between the drill-string and the outer shell was developed by Liao et al. [7]. Later on, Liu et al. [8] studied an eight degrees-of-freedom discrete model taking into account axial, torsional, and lateral dynamics of both the drill-pipes and the BHA. Nandakumar and Wiercigroch [9] considered a fully coupled two degrees-of-freedom model which assumed a state-dependent time delay and a viscous damping for both the axial and torsional motions. In order to study various phenomena, such as stick–slip oscillations, whirling, drill-bit bounce, and helical buckling of the drill-strings, Kapitaniak et al. [2] carried out a comprehensive investigation of a drill-string system including a low-dimensional model of the drilling assembly based on a torsional pendulum and a detailed high-dimensional model of the drilling rig using finite element modeling. In the present work, we will consider the lumped-parameter model shown in Fig. 2, which has been studied in the past by various authors; see, e.g., [10–13]. The generalized lumped-parameter model can be written in a single-input and multi-output form as follows

1 $$\begin{aligned} J\Phi ''+C\Phi '+K\Phi +T=U, \end{aligned}$$

where  is a vector containing the angular positions of the disks, $J=\mathrm {diag}(J_t,$  is the inertia matrix,  is the torsional damping matrix given by

$$\begin{aligned} C=\left[ \begin{array}{ccccccccc} c_p+c_{rt} &{} -c_p &{} 0 &{} 0 &{} \ldots &{} 0 &{} 0 &{} 0 &{} 0 \\ -c_p &{} 2c_p &{} -c_p &{} 0 &{} \ldots &{} 0 &{} 0 &{} 0 &{} 0 \\ 0 &{} -c_p &{} 2c_p &{} -c_p &{} \ldots &{} 0 &{} 0 &{} 0 &{} 0 \\ \ldots &{} \ldots &{} \ldots &{} \ldots &{} \ldots &{} \ldots &{} \ldots &{} \ldots &{} \ldots \\ 0 &{} 0 &{} 0 &{} 0 &{} \ldots &{} -c_p &{} c_p+c_r &{} -c_r &{} 0 \\ 0 &{} 0 &{} 0 &{} 0 &{} \ldots &{} 0 &{} -c_r &{} c_r+c_b &{} -c_b \\ 0 &{} 0 &{} 0 &{} 0 &{} \ldots &{} 0 &{} 0 &{} -c_b &{} c_b+c_{rb} \\ \end{array}\right] , \end{aligned}$$

is the torsional stiffness matrix given by

$$\begin{aligned} K=\left[ \begin{array}{ccccccccc} k_p &{} -k_p &{} 0 &{} 0 &{} \ldots &{} 0 &{} 0 &{} 0 &{} 0 \\ -k_p &{} 2k_p &{} -k_p &{} 0 &{} \ldots &{} 0 &{} 0 &{} 0 &{} 0 \\ 0 &{} -k_p &{} 2k_p &{} -k_p &{} \ldots &{} 0 &{} 0 &{} 0 &{} 0 \\ \ldots &{} \ldots &{} \ldots &{} \ldots &{} \ldots &{} \ldots &{} \ldots &{} \ldots &{} \ldots \\ 0 &{} 0 &{} 0 &{} 0 &{} \ldots &{} -k_p &{} k_p+k_r &{} -k_r &{} 0 \\ 0 &{} 0 &{} 0 &{} 0 &{} \ldots &{} 0 &{} -k_r &{} k_r+k_b &{} -k_b \\ 0 &{} 0 &{} 0 &{} 0 &{} \ldots &{} 0 &{} 0 &{} -k_b &{} k_b \\ \end{array}\right] , \end{aligned}$$

, and , where *u* is the control torque input. Furthermore, $T_b$ is the torque of friction when the drill-bit contacts with the rock, given by

2 $$\begin{aligned} T_b=\left\{ \begin{array}{l l} \tau _r &{} \quad \mathrm {if}\ |\,\phi '_b|\!<\!\zeta \mathrm {and}\ |\,\tau _r|\!\le \tau _s, \\ \tau _s\mathrm {sgn}(\tau _r) &{} \quad \mathrm {if}\ |\,\phi '_b|\!<\!\zeta \mathrm {and}\ |\,\tau _r|\!>\!\tau _s, \\ \mu _b R_b W_{b}\,\mathrm {sgn}(\phi '_b) &{}\quad \mathrm {if}\ |\,\phi '_b|\ge \!\zeta , \end{array} \right. \nonumber \\ \end{aligned}$$

where $\tau _r$ is the reaction torque, $\tau _s$ is the static friction torque, $\zeta >0$ is a small constant, and

$$\begin{aligned} \mu _b=\mu _{cb}+(\mu _{sb}-\mu _{cb})\,e^{-\gamma _b|\phi '_b|/v_f}, \end{aligned}$$

is a velocity-dependent friction coefficient following an exponentially decaying law. Here, $\mu _{cb}$ is the Coulomb friction coefficient, $\mu _{sb}$ is the static friction coefficient, $0<\gamma _b<1$ is a constant defining the velocity decrease rate of $T_b$, $v_f$ is a velocity constant, $R_b$ is the bit radius, and $W_{b}$ is the WOB.

The merit of the model (1) is that the length of the drill-string is taken into consideration, since in practice the drill-string could be several kilometers in length based on the depth of the reservoir. Due to the fact that the diameter of drill-strings typically does not surpass 0.3 m, they are considered highly slender in structure, and particularly, stick–slip oscillations exist in the 50% of drilling time due to the discontinuous friction (2) on the drill-bit. As an underactuated system, the inertia matrix *J* in system (1) is diagonal, while both the damping matrix *C* and the stiffness matrix *K* are symmetric. In [14], this type of systems was classified as flat underactuated systems, and their characteristics were studied by Liu [15].

Currently, there are few experimental investigations for flat multibody underactuated systems in the literature and, similarly, for the case of underactuated systems with discontinuous friction. Therefore, one of the main contributions of the present work is to gain a deeper insight into the dynamics of underactuated systems with discontinuous friction, from an experimental point of view and under realistic conditions, along the lines of, e.g., Kapitaniak et al. [2]. Preliminary studies have been conducted in the past, such as in Melakhessou et al. [16], who carried out an experimental investigation focusing on the local contact between the drill-strings and the well. Mihajlovic et al. [17] used an experimental setup including upper and lower disks to study the friction-induced limit cycles in drill-string systems. A laboratory investigation of drill-string vibrations was carried out by Khulief and AI-Sulaiman [18] by using a magnetic tension brake to simulate stick–slip oscillations. Lu et al. [19] conducted experimental investigations using the D-OSKIL [12] mechanism for mitigation of stick–slip oscillations in oil well drill-strings. An asymmetric vibration damping tool was developed and tested using a small-scale experimental rig in [20]. Patil and Teodoriu [21] conducted a comprehensive comparative review of modeling, control of torsional vibrations, and experimental study of drill-strings under laboratory conditions. Apart from the work of Kapitaniak et al. [2], a common feature of the investigations detailed above is that the experiments which focused on the study of stick–slip oscillations were implemented using disk compression mechanisms to simulate the bit–rock interaction. The experimental study considered in the present paper is based on real rock samples. For this purpose, a small-scale drilling rig has been implemented in order to analyze the stick–slip phenomenon under various control parameters. The rig has been conceived to analyze the dynamics of drilling under realistic conditions, while allowing the variation in various drilling parameters, such as rotary speed and WOB, hence enabling the study of different scenarios of stick–slip oscillations which typically appear in real applications.

The rest of this paper is organized as follows. In Sect. 2, we present a preliminary numerical study of a low-dimensional version of the lumped-parameter model (1). The dynamics of models of this type has been analyzed in the past (see, e.g., [22–24]); however, the numerical investigations of the stick–slip oscillations were carried out using direct numerical integration. In the present work, we will employ path-following (continuation) techniques for non-smooth systems, which will allow us to gain a much deeper insight into the possible control strategies that can be employed to avoid stick–slip oscillations. After this preliminary analysis, we present the experimental apparatus used in the paper in Sect. 3. The identification of the drill-string parameters including the damping constants, the spring constants, and the torsion constants of two flexible shafts used in the experimental rig is carried out in Sect. 4. The main experimental results are presented and discussed in Sect. 5. The paper finalizes with some concluding remarks and an outlook for future work.

## A preliminary numerical study of stick–slip oscillations

### Mathematical model

In this section, we will consider a particular case of the general model shown in Fig. 2. This picture shows a physical representation of a drilling system based on a series of disks interconnected via torsional springs and dampers. For our numerical study, we will consider a model with four disks, corresponding to the rotary table (*t*), drill-pipe (*p*), drill-collar (*r*) and drill-bit (*b*). Due to the stick and slip phases that can take place in the considered drilling scenario, the modes of operation of the system can be divided into two cases as follows:

*Slip (SL)*. In this mode, the drill-bit rotates with positive angular velocity, and the system motion is governed by Eq. [cf. (1)]

3 $$\begin{aligned} \left\{ \begin{aligned}&J_{t}\phi ''_{t}+c_{rt}\phi '_{t}+c_{p}(\phi '_{t}-\phi '_{p})+k_{p}(\phi _{t}-\phi _{p})=u,\\&J_{p}\phi ''_{p}\!+c_{p}(\phi '_{p}\!-\phi '_{t})\!+c_{r}(\phi '_{p}\!-\phi '_{r})\!+k_{p}(\phi _{p}\!-\phi _{t})\!+k_{r}(\phi _{p}-\phi _{r})=0,\\&J_{r}\phi ''_{r}\!+c_{r}(\phi '_{r}\!-\phi '_{p})\!+\!c_{b}(\phi '_{r}\!-\phi '_{b})\!+k_{r}(\phi _{r}-\phi _{p})\!+k_{b}(\phi _{r}\!-\phi _{b})=0,\\&J_{b}\phi ''_{b}+c_{rb}\phi '_{b}+c_{b}(\phi '_{b}-\phi '_{r})+k_{b}(\phi _{b}-\phi _{r})+T^{\text {SL}}_{b}=0, \end{aligned}\right. \nonumber \\ \end{aligned}$$

where $\phi _{t}$, $\phi _{p}$, $\phi _{r}$, and $\phi _{b}$ give the angular position of the rotary table, drill-pipe, drill-collar, and drill-bit, respectively. Furthermore, the reaction torque is computed as

4 $$\begin{aligned} T^{\text {SL}}_{b}= {\left\{ \begin{array}{ll}T_{0}, &{}\!\! \omega _{b}\!\!=\!0,\\ R_{b}W_{b}\left( \mu _{cb}+(\mu _{sb}-\mu _{cb})e^{-\lambda _{b}\omega _{b}}\right) , &{}\!\! \omega _{b}\!\!>\!0, \end{array}\right. }\nonumber \\ \end{aligned}$$

which is a simplified version of the torque function shown in (2). In the expression above, $\omega _{b}$ is the angular velocity of the drill-bit (i.e., $\omega _{b}=\phi '_{b}$) and $T_{0}=\mu _{sb}R_{b}W_{b}$ is the break-away torque. This operation mode terminates at some $t=t_{\text {stick}}\ge 0$ when the angular speed of the drill-bit becomes zero, i.e., $\omega _{b}(t_{\text {stick}})=0$. At this time, the system switches to the stick mode of operation defined below.

*Stick (ST)*. In this phase, the drill-bit is in stationary position, and the dynamics of the model is described by the system

5 $$\begin{aligned} \left\{ \begin{aligned}&J_{t}\phi ''_{t}\!+c_{rt}\phi '_{t}\!+c_{p}\left( \phi '_{t}-\phi '_{p}\right) \!+k_{p}\left( \phi _{t}\!-\phi _{p}\right) =u,\\&J_{p}\phi ''_{p}\!+\!c_{p}\left( \phi '_{p}\!-\phi '_{t}\right) \!+\!c_{r}\left( \phi '_{p}\!-\phi '_{r}\right) \!+\!k_{p}\left( \phi _{p}\!-\!\phi _{t}\right) \!+\!k_{r}\left( \phi _{p}\!-\!\phi _{r}\right) \!=\!0,\\&J_{r}\phi ''_{r}\!+c_{b}\phi '_{r}+c_{r}\left( \phi '_{r}-\phi '_{p}\right) +k_{r}\left( \phi _{r}-\phi _{p}\right) +k_{b}\left( \phi _{r}-\phi _{b}\right) =0,\\&\phi ''_{b}=0,\,\,\,\phi '_{b}=0. \end{aligned}\right. \nonumber \\ \end{aligned}$$

During this mode, the reaction torque is computed via Newton’s third law as follows

6 $$\begin{aligned} T^{\text {ST}}_{b}=c_{b}\phi '_{r}+k_{b}(\phi _{r}-\phi _{b}), \end{aligned}$$

which means that the reaction torque adjusts itself to enforce the equilibrium with the external torque acting on the drill-bit. This mode terminates at some $t=t_{\text {slip}}\ge 0$ when $\left. T^{\text {ST}}_{b}\right| _{t=t_{\text {slip}}}=T_{0}$. At this point, the reaction torque has reached the break-away torque value $T_{0}$, where the drill-bit begins to rotate, hence switching the system to the slip phase introduced previously.

In order to numerically study periodic and equilibrium solutions of the drill-string model (3)–(6), it is convenient to introduce the following variable transformation

7 $$\begin{aligned} \left\{ \begin{aligned} x_{t}&=\phi _{t},\\ y_{t}&=\omega _{t},\\ x_{p}&=\phi _{p}-\phi _{t},\\ y_{p}&=\omega _{p},\\ x_{r}&=\phi _{r}-\phi _{p},\\ y_{r}&=\omega _{r},\\ x_{b}&=\phi _{b}-\phi _{r},\\ y_{b}&=\omega _{b},\\ \end{aligned}\right. \end{aligned}$$

where $\omega _{t}$, $\omega _{p}$, $\omega _{r}$, and $\omega _{b}$ give the angular velocity of the rotary table, drill-pipe, drill-collar, and drill-bit, respectively. According to the operation regimes defined above, the equations of motion of the system can be written in compact form in terms of the new variables as follows

8 $$\begin{aligned} z'= {\left\{ \begin{array}{ll} f_{\text {ST}}(z,\alpha ), &{} y_{b}\!=0 \text{ and } T^{\text {ST}}_{b}\!=\!c_{b}y_{r}\!-\!k_{b}x_{b}\!<\!T_{0},\\ f_{\text {SL}}(z,\alpha ), &{} \text{ otherwise }, \end{array}\right. }\nonumber \\ \end{aligned}$$

where  and  stand for the parameters and state variables of the piecewise smooth system, respectively. In the system introduced above, the vector fields $f_{\text {ST}}$ (stick) and $f_{\text {SL}}$ (slip) are defined as [cf. (3), (5) and (7)]

$$\begin{aligned} f_{\text {ST}}(z,\alpha )&=\left( \begin{array}{l} \frac{1}{J_{t}}\left( u-c_{rt}y_{t}+k_{p}x_{p}+c_{p}(y_{p}-y_{t})\right) \\ y_{p}-y_{t}\\ \frac{1}{J_{p}}\left( k_{r}x_{r}-k_{p}x_{p}+c_{p}(y_{t}-y_{p})+c_{r}(y_{r}-y_{p})\right) \\ y_{r}-y_{p}\\ \frac{1}{J_{r}}\left( k_{b}x_{b}-k_{r}x_{r}-c_{b}y_{r}+c_{r}(y_{p}-y_{r})\right) \\ -y_{r}\\ 0 \end{array}\right) ,\\ f_{\text {SL}}(z,\alpha )&=\left( \begin{array}{l} \frac{1}{J_{t}}\left( u-c_{rt}y_{t}+k_{p}x_{p}+c_{p}(y_{p}-y_{t})\right) \\ y_{p}-y_{t}\\ \frac{1}{J_{p}}\left( k_{r}x_{r}-k_{p}x_{p}+c_{p}(y_{t}-y_{p})+c_{r}(y_{r}-y_{p})\right) \\ y_{r}-y_{p}\\ \frac{1}{J_{r}}\left( k_{b}x_{b}-k_{r}x_{r}+c_{b}(y_{b}-y_{r})+c_{r}(y_{p}-y_{r})\right) \\ y_{b}-y_{r}\\ \frac{1}{J_{b}}\left( c_{b}(y_{r}-y_{b})-c_{rb}y_{b}-k_{b}x_{b}-T^{\text {SL}}_{b}\right) \end{array}\right) . \end{aligned}$$

### Numerical investigation of the drill-string model

In this section, we will present a detailed numerical investigation of the dynamical response of the drill-string model (8). For this purpose, we will apply numerical continuation methods for non-smooth dynamical systems, implemented via the continuation platform COCO [25, 26]. The results of the numerical investigation will be presented using the following solution measure

9 $$\begin{aligned} M_{\omega _{b}}=\frac{1}{T_{0}}\int \limits _{0}^{T_{0}}y_{b}(t)\,\mathrm{d}t=\frac{1}{T_{0}}\int \limits _{0}^{T_{0}}\omega _{b}(t)\,\mathrm{d}t, \end{aligned}$$

which gives the average angular velocity of the drill-bit in the time window $[0,T_{0}]$, where $T_{0}$ is a suitably chosen positive number. In the case of studying periodic orbits, $T_{0}$ will correspond to the period of the solution.

**Fig. 3:**
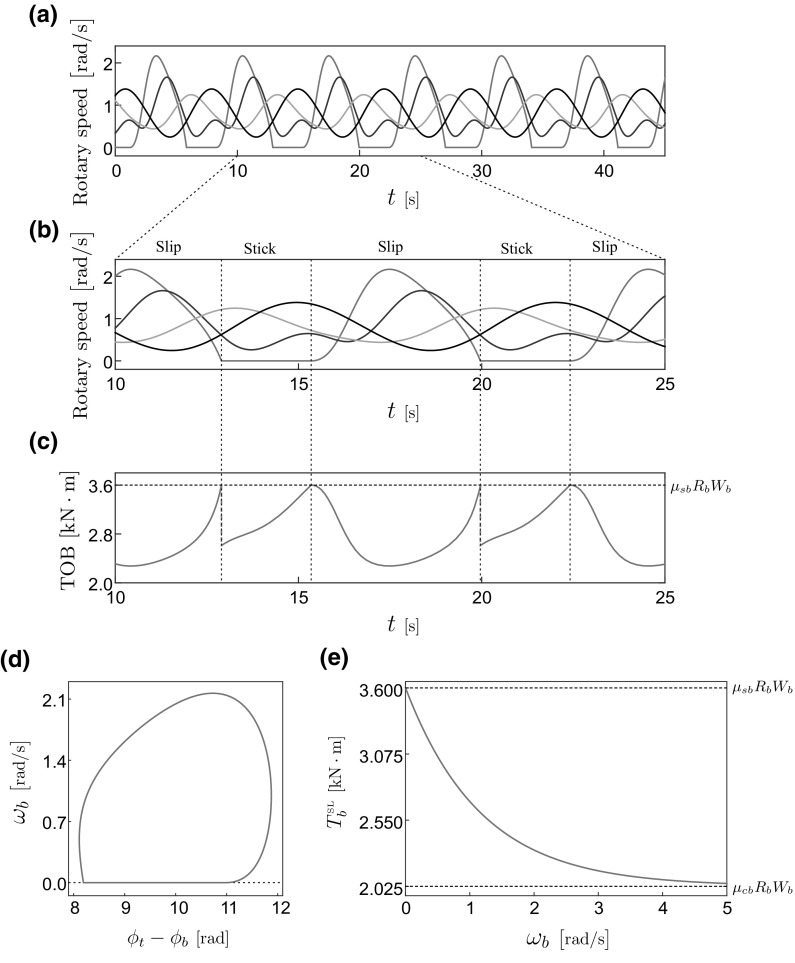
Stick–slip behaviour obtained from the piecewise smooth model (8), for the parameter values $J_{t}=910\,\text {kg}\,\text {m}^2$, $J_{p}=2800\,\text {kg}\,\text {m}^2$,  $J_{r}=750\,\text {kg}\,\text {m}^2$, $J_{b}=450\,\text {kg}\,\text {m}^2$,  $c_{rt}=410\,\text {Nm}\,\text {s}/\text {rad}$,  $c_{p}=150\,\text {Nm}\,\text {s}/\text {rad}$,  $c_{r}=190\,\text {Nm}\,\text {s}/\text {rad}$,  $c_{b}=180\,\text {Nm}\,\text {s}/\text {rad}$,  $c_{rb}=80\,\text {Nm}\,\text {s}/\text {rad}$,  $k_{p}=700\,\text {Nm}/\text {rad}$,  $k_{r}=1080\,\text {Nm}/\text {rad}$,  $k_{b}=910\,\text {Nm}/\text {rad}$, $R_{b}=0.15\,\text {m}$,  $W_{b}=3\times 10^4\,\text {N}$,  $\lambda _{b}=0.85\,\text {s}/\text {rad}$, $u=3200\,\text {Nm}$, $\mu _{cb}=0.45$ and $\mu _{sb}=0.8$. **a** Time response of the model showing the angular velocities of the drill-bit ($\omega _{b}$, in blue), collar ($\omega _{r}$, in red), pipe ($\omega _{p}$, in green), and rotary table ($\omega _{t}$, in black). **b** Segment of the time response shown in (**a**) for the time window $10\le t\le 25$. **c** Torque on bit observed during operation. **d** Phase diagram corresponding to the periodic solution depicted in (**a**). **e** Reaction torque as a function of the angular velocity, computed from (4). (Color figure online)

**Fig. 4:**
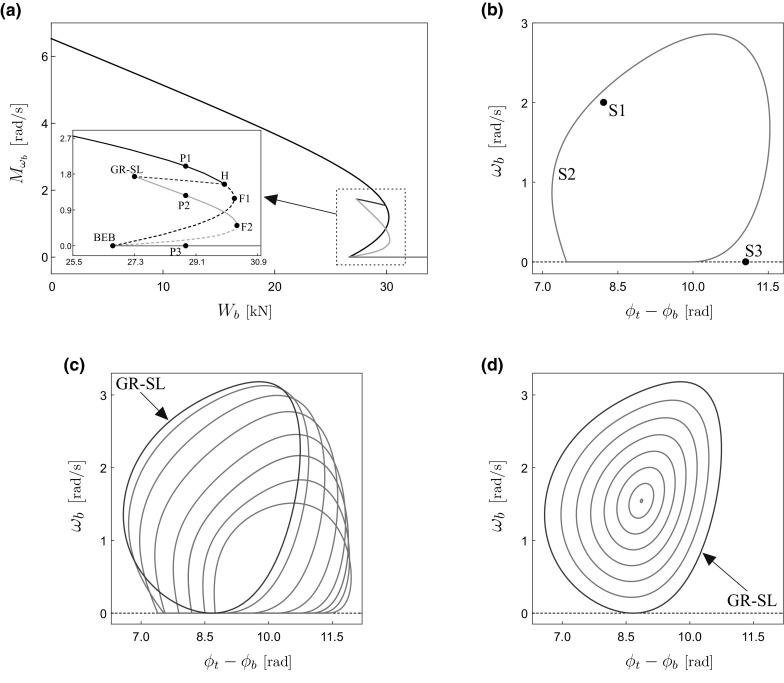
**a** Numerical continuation of the dynamical response of the piecewise smooth model (8) with respect to the WOB $W_{b}$. The black and blue curves represent the continuation of equilibrium points corresponding to $\omega _{b}>0$ (constant rotation) and $\omega _{b}=0$ (permanent sticking), respectively. The green curve stands for the continuation of stick–slip solutions (as shown in Fig. 3), while the red branch corresponds to periodic solutions with no sticking phases. Solid and dashed lines denote stable and unstable solutions, respectively. The vertical axis gives the average angular speed of the drill-bit computed via (9). **b** Phase portrait showing three coexisting attractors: S1 (equilibrium with $\omega _{b}>0$), S2 (stick–slip solution), and S3 (equilibrium with $\omega _{b}=0$) corresponding to the test points P1, P2, and P3, respectively, shown in panel (**a**) with $W_{b}=28.8$ kN. Panels **c**, **d** depict periodic solutions of system (8) along the green and red branches shown in panel (**a**), around the grazing–sliding bifurcation GR-SL. The grazing–sliding solution is plotted with red color. (Color figure online)

**Fig. 5:**
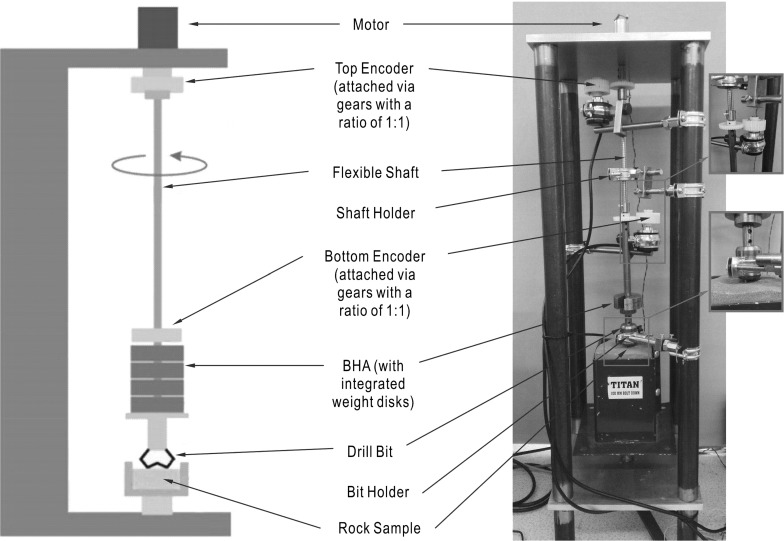
Schematic diagram of the experimental setup (left) and photograph of the experimental rig (right)

An initial periodic response is shown in Fig. 3, which corresponds to a cyclic stick–slip behavior of the drill-string model (8). In panel (b), we show the periodic response for the time window $10\le t\le 25$s. In the time interval $10\le t<t_{\text {stick}}\approx 12.90$s, the angular velocity of the drill-bit $\omega _{b}$ is positive, and hence, the system operates under the slip mode defined in the previous section. At $t=t_{\text {stick}}$, $\omega _{b}$ becomes zero, and thus, the system switches to the stick operation mode. Due to the sudden jump of the angular acceleration of the drill-bit from a negative value to zero, the torque on bit presents a discontinuity at $t=t_{\text {stick}}$; see Fig. 3c. As the angular displacement $\phi _{t}$ of the rotary table increases, the reaction torque $T^{\text {ST}}_{b}$ grows due to the increasing elastic energy stored in the torsional spring $k_{b}$; see (6). The stick regime finishes when the break-away torque value $T_{0}=\mu _{sb}R_{b}W_{b}$ is reached at $t=t_{\text {slip}}\approx 15.35$s, where the system changes to the slip mode of operation and remains there as long as the angular velocity of the drill-bit is positive. Panel (d) in Fig. 3 shows the phase portrait corresponding to the stick–slip solution, and panel (e) shows the behaviour of the torque function (4) used for the numerical study.

It is worth noting that, as shown in Fig. 3d, a horizontal shift of the sliding phase, i.e., the upper maximum of the presented trajectory shifted to the higher relative angular displacement $\phi _t-\phi _b$, can be clearly observed on the phase trajectory. Similar shifting phenomenon can be found from the work [27], where a self-excited friction pair was investigated numerically and experimentally. The reason for the shifting to the right is because of the frictional torque considered in our investigation, which leads to an asymmetry in the way the orbit enters and leaves the sticking phase. Looking at the stick–slip orbit shown in Fig. 3d, it can be observed that when the solution enters the sticking phase, the velocity is not differentiable. This is explicitly observed in the time history given in Fig. 3b plotted in blue. This is because the torque on bit drops suddenly at this point, and hence, the acceleration presents a jump, due to which the velocity exhibits a singularity. This effect is due to the friction, which is known to affect reversibility, that is, the way the model enters the stick regime does not coincide with the path the model leaves such operation mode. On the other hand, when the system leaves the sticking phase, there is a smooth transition owing to the continuity of the torque on bit at the transition point and hence of the acceleration. That is why the velocity is differentiable at this point, which can be clearly seen in the stick–slip orbit presented in Fig. 3d [see also panel (b)]. Therefore, at the point where the drill-bit’s velocity becomes zero, there is a sharp transition in the orbit, whereas the switching to the slip phase is smooth. This produces the resulting shifting to the right of the orbit.

The result of the numerical continuation of the dynamical response of the drill-string model (8) with respect to the WOB $W_{b}$, showing the average angular speed of the drill-bit [see (9)] on the vertical axis, is presented in Fig. 4. In this figure, stable and unstable solutions are represented by solid and dashed lines, respectively. The continuation was carried out starting from $W_{b}=0$ (no WOB). As physically expected, in this case all four disks rotate with a constant angular speed, which is represented by the system as a stable equilibrium. This stable equilibrium persists for larger values of $W_{b}$, and the continuation of such points is depicted in Fig. 4a as a solid black curve. The stability of the equilibrium, however, is lost at the point H ($W_{b}\approx 29.9$ kN), where the system undergoes a subcritical Hopf bifurcation, corresponding to a so-called catastrophic loss of stability (see [28, Section 3.4]), characterized by a positive first Lyapunov coefficient at the bifurcation point. An unstable branch of equilibria can be traced after the Hopf bifurcation (dashed black curve), until a fold bifurcation (labeled F1) at $W_{b}\approx 30.2$ kN is found. Here, the branch of unstable equilibria makes a “turn” toward the decreasing direction of the parameter $W_{b}$. As $W_{b}$ becomes smaller, the velocity of the drill-bit $\omega _{b}$ also reduces, reaching the critical value $\omega _{b}=0$ at $W_{b}\approx 26.7$ kN). Here, a boundary-equilibrium bifurcation (labeled BEB) takes place, consisting in a situation in which an equilibrium point lies at a discontinuity boundary (see [29, Section 5.1]). Such bifurcations can manifest themselves in several ways, for instance as a non-smooth fold, as it is in our case. In this scenario, we have that a branch of unstable (dashed black curve) and stable (solid blue curve) equilibria collide at the BEB point, hence resembling the behaviour observed for fold bifurcations of smooth systems. The blue curve corresponds to the continuation of stable equilibrium points for which the drill-bit does not rotate (permanent sticking). This system response persists for increasing values of WOB, which is physically explained in terms of the increasing break-away torque $T_{0}=\mu _{sb}R_{b}W_{b}$ that needs to be overcome in order to switch the system to the slip mode of operation.

**Fig. 6:**
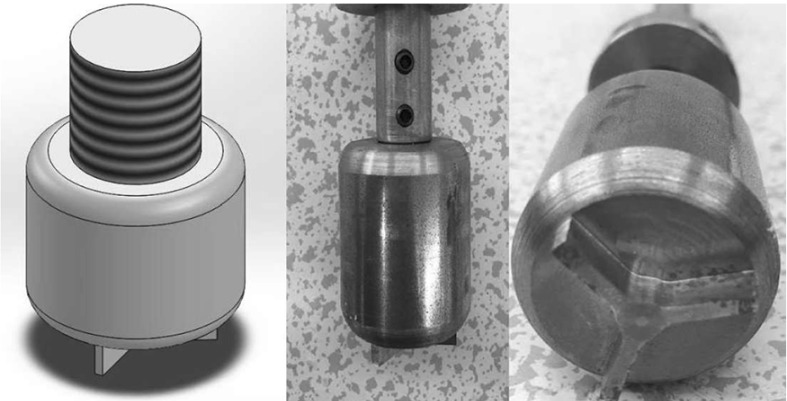
Drill-bit Solidworks drawing (left), photograph of the drill-bit (middle), and photograph of the drill-bit’s tri-blade design (right)

**Fig. 7:**
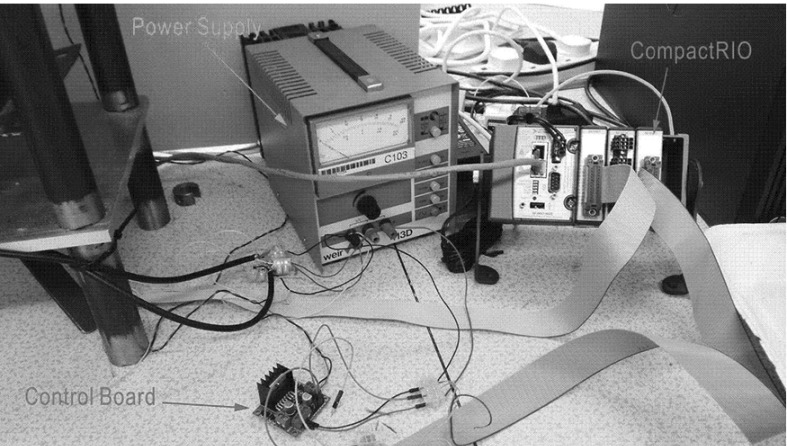
Photograph of the CompactRIO (data acquisition device) and motor control system

**Fig. 8:**
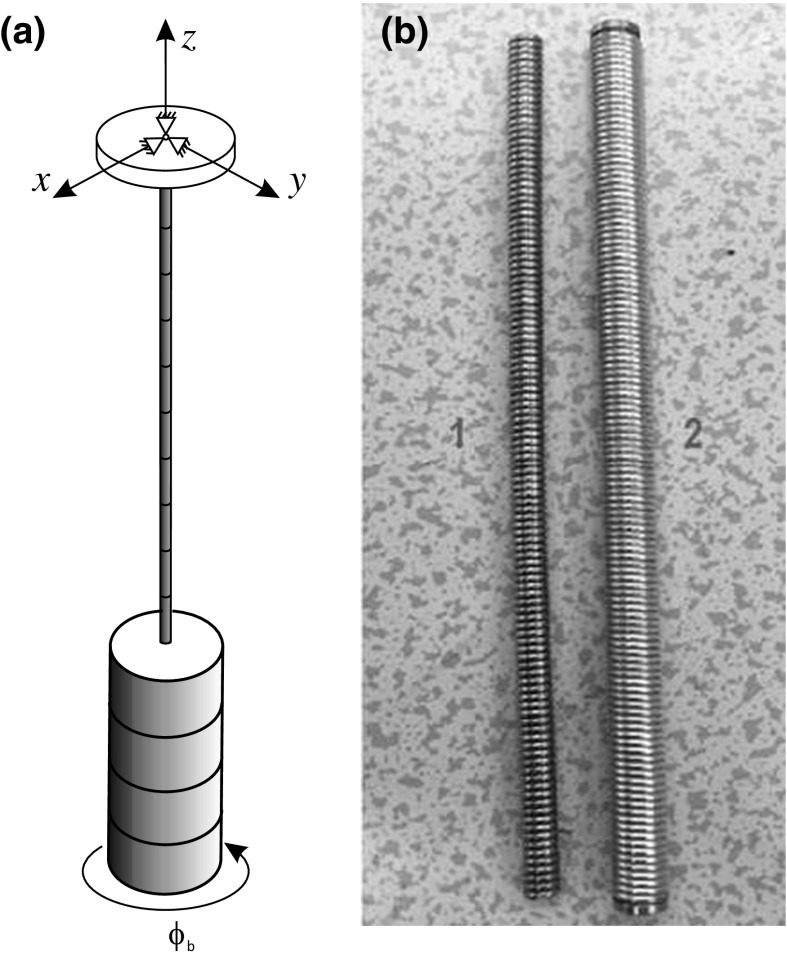
**a** Setup of the apparatus used to determine the damping and the torsion stiffness of the flexible shafts (adopted from [2]) and **b** photograph of the two flexible shafts

At the BEB point found before there is an emanating curve of unstable periodic orbits (plotted as a dashed green line), which is a typical scenario encountered for boundary-equilibrium bifurcations; see, e.g., [29, Section 5.1.2]. If we increase the parameter $W_{b}$ along the green curve, a fold bifurcation of limit cycles is encountered for $W_{b}\approx 3.03\times 10^4\,\text {N}$ (labeled F2). Here, a branch of unstable and a branch of stable periodic solutions collide, both of them corresponding to periodic solutions showing stick–slip oscillations. The stable periodic orbits persist for decreasing values of $W_{b}$, until the critical point $W_{b}\approx 37.3$ kN is reached. At this point, a grazing–sliding bifurcation (labeled GR-SL) of limit cycles takes place. The evolution of the stick–slip solutions toward the grazing–sliding solution is observed in Fig. 4c. The grazing–sliding bifurcation found in the drill-string model (8) is characterized by a so-called non-smooth fold transition; see [29, Section 8.5.3]. This is because, similarly as in the scenario described in the previous paragraph, a branch of stable and a branch of unstable periodic solutions collide at the GR-SL point. The unstable branch is plotted as a dashed red line in panel (a), and the evolution of the periodic orbits along this branch can be seen in panel (d). This dashed line can be traced for increasing values of $W_{b}$, until the critical point H (found before) is reached. This means that the branch of unstable periodic solutions is created at the subcritical Hopf bifurcation encountered at $W_{b}\approx 29.9$ kN.

**Fig. 9:**
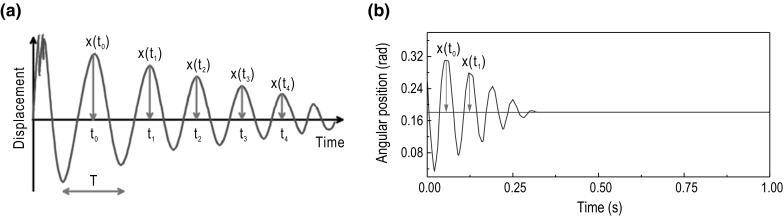
**a** Example graph of decaying free torsional oscillations and **b** the sample graph of free torsional decaying oscillations for shaft 1, where *x*(*t*) is the angular position of the shaft in radians

Another important feature of the bifurcation scenario observed in the system is that there is a parameter window defined by the GR-SL and the H points found before in which three attractors coexist; see Fig. 4b. The first attractor (labeled S1 in the picture) corresponds to a stable equilibrium with constant drill-bit rotation, which can be identified as a desirable solution, from a practical point of view. The orbit S2 represents a stable stick–slip solution that lies on the solid green curve shown in Fig. 4a. The third attractor S3 is found on the horizontal blue line, which represents an equilibrium solution with permanent sticking. Therefore, in this parameter window the system presents multistability, a feature that can be used to steer the response from, e.g., a sticking equilibrium or stick–slip oscillation to an equilibrium with constant drill-bit rotation.

This preliminary numerical study shows in great detail the important role played by the WOB in order to control the dynamical behaviour of the considered drilling system. The remaining sections of the paper will be devoted to the experimental study of stick–slip oscillations based on real rock drilling, and one of the main issues to be investigated will be precisely the control of undesired oscillations using the WOB as control parameter.

## Experimental investigation of stick–slip oscillations

### Rig description

The main concern in the experimental part of the present paper will be to study in detail the phenomenon of stick–slip and how to avoid this undesired behaviour by adjusting system parameters, such as the WOB, which was proved to play a fundamental role in the drill-string dynamics (see previous section). Therefore, the proposed rig design is focused on studying the rotational motion of a small-scale drill-string, where axial and lateral vibrations are restricted. The rig has a modular design whereby two flexible shafts with varying degrees of stiffness can be used. The total dimensions of the rig’s casing are $84.5\times 25\times 25$ cm, and the main components of the experimental rig are outlined in Fig. 5, where rotary motion is supplied to the drill-string through a 12V DC electrical motor, located at the top of the casing with speed ranging from 0 to 120 rpm. The drill-pipe is represented by an interchangeable steel flexible shaft which transmits the rotary motion to the BHA from the motor. The mechanical characteristics of the two flexible shafts used in this experimental rig will be discussed in further detail later in this section. The BHA is comprised of a series of short steel cylinders with varying mass, as shown in Fig. 5, whereby the steel cylinders act as adjustable weight disks which are used to control the WOB of the drill-string.

**Table 1:** Shaft parameters and spring constants

Shaft	1	2
Mean coil diameter, *D* (mm)	9.10	5.16
Number of coils, *N*	119.00	116.00
Wire diameter, *d* (mm)	1.6	1.64
Shear modulus of shaft, *G* (GPa)	68.50	68.50
Mean coil radius, *R* (mm)	4.55	2.58
Spring constant, *k* (N/m)	616.24	3699.73

**Table 2:** Torsion constants and frictional torque

Shaft	1	2
Mean coil radius, *R* (mm)	4.55	2.58
Torsion constant, $\kappa$ ($\mathrm {m}^4$)	$1.28\times 10^{-2}$	$2.46\times 10^{-2}$
Angle of twist 1, $\theta ^1$, ($\circ$)	180	94
Frictional torque 1, $T_b^1$, (Nm)	$-$ 2.30	$-$ 2.31
Angle of twist 2, $\theta ^2$, ($\circ$)	460	239
Frictional torque 2, $T_b^2$, (Nm)	$-$ 5.89	$-$ 5.88
Angle of twist 3, $\theta ^3$, ($\circ$)	720	375
Frictional torque 3, $T_b^3$, (Nm)	$-$ 9.22	$-$ 9.23

**Table 3:** Parameters of the drill-string rig for experimental tests

Test no.	Motor rotary speed (rad/s)	Frictional torque, $T_b$ (Nm)	Damping constant, $\zeta$($\times 10^{-2}$ in J s/rad)	Spring constant, *k* (N/m)	Torsion constant, $\kappa$($\times 10^{-2}$ in $\mathrm {m}^4$)
1(a)	2.7	$-$ 2.30	1.91	616.24	1.28
1(b)	5.3	$-$ 2.30	1.91	616.24	1.28
2(a)	2.7	$-$ 5.89	1.91	616.24	1.28
2(b)	5.3	$-$ 5.89	1.91	616.24	1.28
3(a)	2.7	$-$ 9.22	1.91	616.24	1.28
3(b)	4.6	$-$ 9.22	1.91	616.24	1.28
4(a)	2.7	$-$ 2.31	1.32	3699.73	2.46
4(b)	5.3	$-$ 2.31	1.32	3699.73	2.46
5(a)	2.7	$-$ 5.88	1.32	3699.73	2.46
5(b)	5.3	$-$ 5.88	1.32	3699.73	2.46
6(a)	2.7	$-$ 9.23	1.32	3699.73	2.46
6(b)	4.6	$-$ 9.23	1.32	3699.73	2.46

**Fig. 10:**
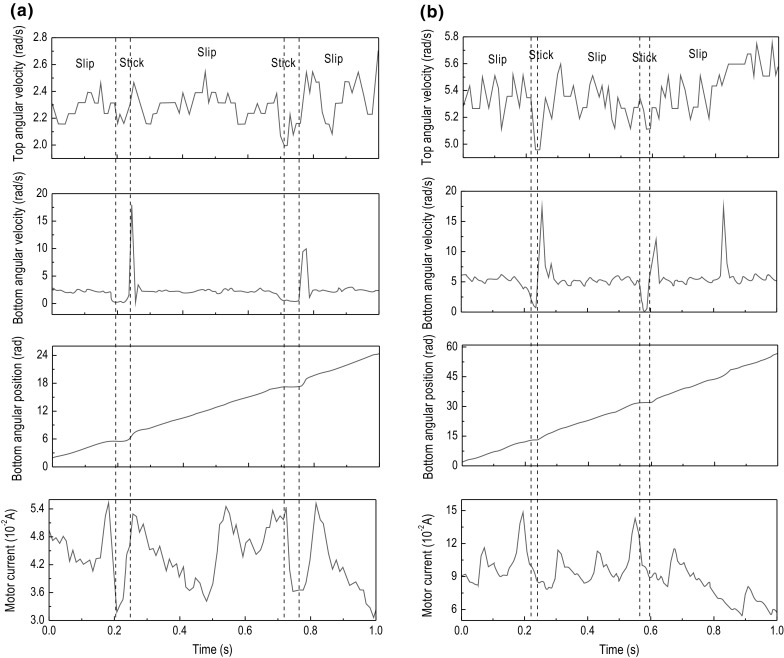
Experimental results of Test 1. The time histories of top angular velocity, bottom angular velocity, bottom angular position, and motor current are presented for $\kappa =1.28\times 10^{-2}\, \mathrm {m}^4$, $k=616.24$ N/m, $\zeta =1.91\times 10^{-2}$ J s/rad, and $T_b=-2.3$ Nm obtained at the motor speed of **a** 2.7 rad/s and **b** 5.3 rad/s

**Fig. 11:**
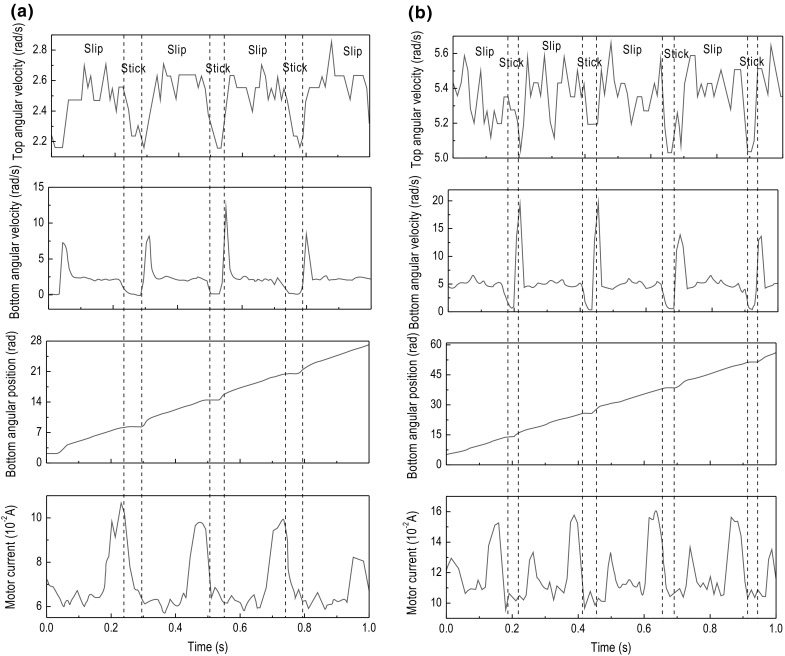
Experimental results of Test 2. The time histories of top angular velocity, bottom angular velocity, bottom angular position, and motor current are presented for $\kappa =1.28\times 10^{-2}\, \mathrm {m}^4$, $k=616.24$ N/m, $\zeta =1.91\times 10^{-2}$ J s/rad, and $T_b=-5.89$ Nm obtained at the motor speed of **a** 2.7 rad/s and **b** 5.3 rad/s

**Fig. 12:**
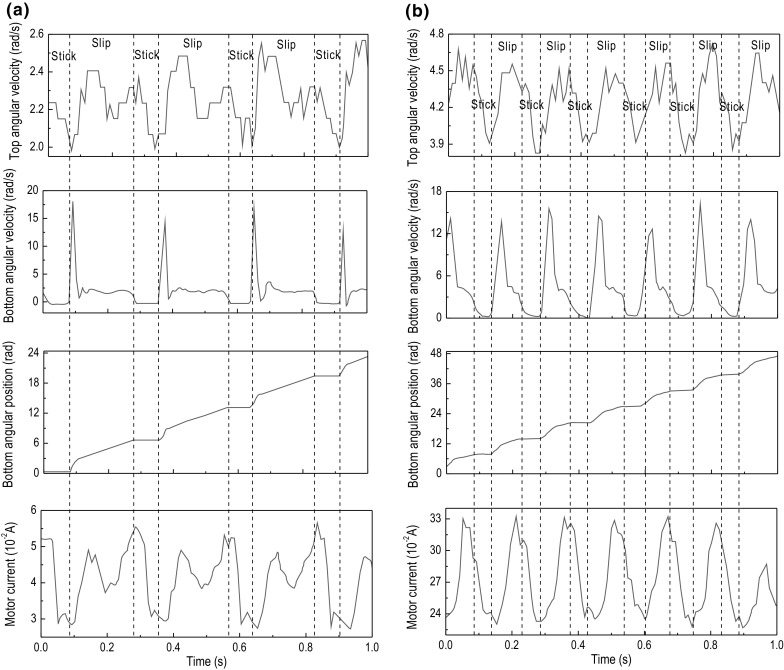
Experimental results of Test 3. The time histories of top angular velocity, bottom angular velocity, bottom angular position, and motor current are presented for $\kappa =1.28\times 10^{-2}\, \mathrm {m}^4$, $k=616.24$ N/m, $\zeta =1.91\times 10^{-2}$ J s/rad, and $T_b=-9.22$ Nm obtained at the motor speed of **a** 2.7 rad/s and **b** 4.6 rad/s

**Fig. 13:**
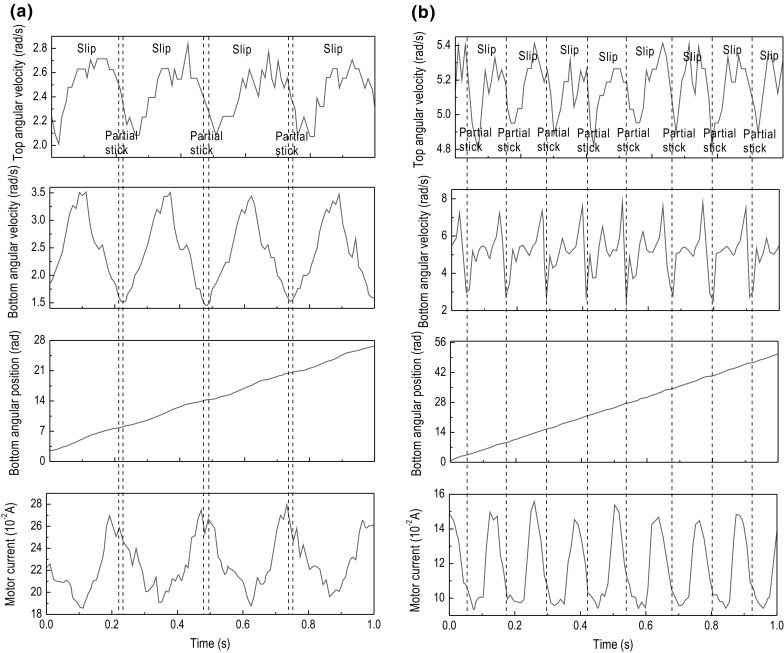
Experimental results of Test 4. The time histories of top angular velocity, bottom angular velocity, bottom angular position, and motor current are presented for $\kappa =2.46\times 10^{-2}\, \mathrm {m}^4$, $k=3699.73$ N/m, $\zeta =1.32\times 10^{-2}$ J s/rad, and $T_b=-2.31$ Nm obtained at the motor speed of **a** 2.7 rad/s and **b** 5.3 rad/s

**Fig. 14:**
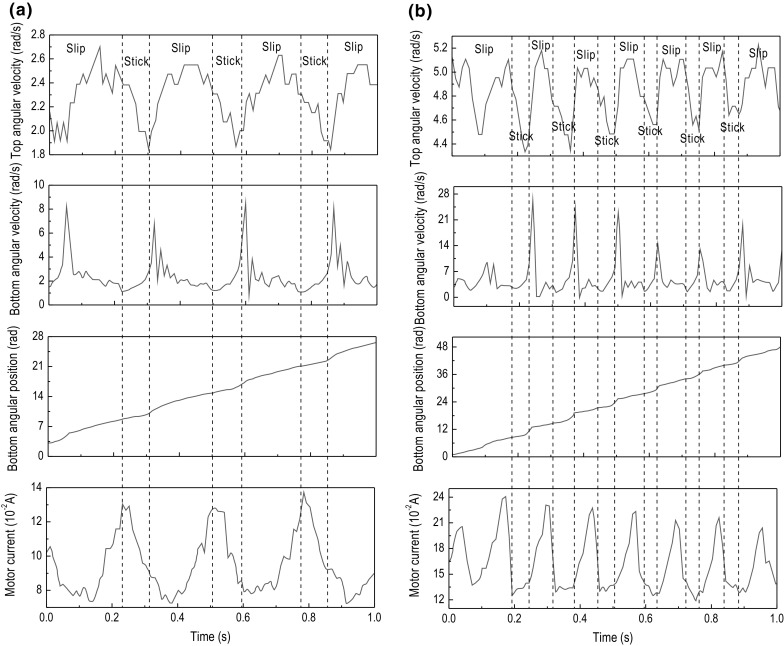
Experimental results of Test 5. The time histories of top angular velocity, bottom angular velocity, bottom angular position, and motor current are presented for $\kappa =2.46\times 10^{-2}\, \mathrm {m}^4$, $k=3699.73$ N/m, $\zeta =1.32\times 10^{-2}$ J s/rad, and $T_b=-5.88$ Nm obtained at the motor speed of **a** 2.7 rad/s and **b** 5.3 rad/s

A tri-blade design as shown in Fig. 6 has been implemented for the drill-bit, and dry sand was initially used as the test sample in the experimental rig in order to provide the required amount of friction to produce the stick–slip phenomenon. However, after conducting the first set of tests, it was discovered that dry sand was incapable of providing enough friction for the drill-bit to enter the stick phase. Therefore, the experimental setup was adjusted so as to use real rock samples in the drilling tests, where a rotary handle under the experimental rig was designed to change the height of the sample in order to provide extra WOB for the drill-string.

Two identical quadrature rotary encoders were used in the experimental rig to measure the angular velocities at the top and bottom of the drill-string. The encoders were incorporated into the system through a set of gears meshed together with a 1:1 ratio as shown in the blowup window in Fig. 5. The signals from the rotary encoders were transmitted to the CompactRIO, a data acquisition device as shown in Fig. 7. The CompactRIO was also implemented to monitor the voltage and current levels of the motor through the control board, and the motor was controlled by a LabVIEW graphical interface which allowed the real-time response from the experimental rig to be monitored.

## Parameter identification

When conducting experimental investigations, appropriate estimations of the physical parameters in the experimental setup needed to be determined, in order to keep the physical characteristics of the rig under meaningful ranges. Therefore, a series of tests were conducted on the rig to experimentally determine some of its physical parameters, with a main focus on the drill-string. These include determination of the damping constants, the spring constant, and the torsion constants of the flexible shafts. In the literature, flexible shafts are seen to be used to simulate the mechanical characteristics of slender structures, such as drill-strings [2]. As previously stated, drill-strings are typically known to have lengths of up to several kilometers long and so they can be considered to have a minuscule amount of transversal stiffness in comparison with that in the axial direction. This is commonly seen to be represented on a small scale by a flexible shaft with multiple layers of thin wire. However, in this investigation the flexible shafts used were long slender helical springs with closely packed coils. These were deemed as acceptable substitutes to the ones commonly used as they have a relatively high levels of flexibility and allow for high torque capacity transmission.

In Fig. 8b, we present a photograph of the two flexible shafts used in the experimental investigation, which have diameters of 9.10 and 5.16 mm, both with a length of 191.00 mm. The values of viscous damping exhibited in the two flexible shafts were determined through a standard mathematical procedure implemented via the system design platform LabVIEW. By applying an angular displacement, $\phi _b$, to the BHA and therefore the shaft, a measurement of the decaying free torsional oscillations could be taken. Figure 8a shows a schematic representation of the experimental setup, and Fig. 9a presents an example of the typical waveform exhibited when the test was conducted. From the signals obtained, the damping constant (denoted hereafter by the parameter $\zeta$) can be estimated by applying standard fitting procedures using an exponentially damped sinusoidal function. In order to ensure confidence in the parameter estimations, the free oscillation test was repeated ten times for each shaft, and thus, the average values $\zeta _1=1.91\times 10^{-2}$ and $\zeta _2=1.32\times 10^{-2}\, \mathrm {J}\,\mathrm {s/rad}$ were obtained (see Fig. 9b for a sample graph of the tests).

According to [30], it is found that for a close-coiled helical spring, the spring constant, *k*, of each flexible shaft can be determined by the formula

$$\begin{aligned} k=\frac{Gd^4}{8ND^3}\left[ 1+\frac{1}{2}\left( \frac{d}{D}\right) ^2\right] ^{-1}, \end{aligned}$$

where the first and second terms in the square brackets correspond to the effects of torsion and pure shear, respectively, *N* is the numbers of turns in the coils of the spring, *d* is the diameter of the wire, *D* is the mean diameter of the coils, and *G* is the shear modulus of the material. The wires in the two flexible shafts are constructed from a stainless steel material and therefore have a shear modulus $G=68.5$ GPa [31]. The spring constants of the two flexible shafts were calculated, and their parameters are presented in Table 1.

To study some of the characteristics of the bit–rock interactions in downhole drilling, the varying frictional torque on the drill-bit in regards to stick–slip was investigated. Due to some rig limitations, it was not possible to use a torque measuring device for this purpose. Hence, the torque exhibited on the drill-bit due to friction, $T_b$, during the testing was determined experimentally as follows. According to Hooke’s Law, the restoring (reaction) torque is proportional to the angle of twist, $\theta$, given as $\tau =-\kappa \,\theta$, where $\kappa$ is the torsion constant of the wire and $\theta$ is the angle of twist applied to the spring. There are many ways in which the torsion constant $\kappa$ could be determined experimentally and theoretically. A universal relationship exists that can be used to establish a link between the torsion constant of a wire and the spring constant of a close-coiled helical spring, which is wound from the same wire. An experimental study by Mohazzabi and Shefchik [30] verifies this relationship, according to the formula

$$\begin{aligned} \kappa =R^2k, \end{aligned}$$

where *R* is the mean coil radius of the helical spring. It should be noted that this relationship is only valid in this form for ordinary springs where the ratio of the diameter of the wire *d* to the diameter of the coil *D* is less than 1, i.e., $\tfrac{d}{D}<1$. The flexible shafts used in our experimental tests were chosen so as to satisfy this condition, and therefore, the formula above was employed to determine the torsion constants of the shafts which is found in Table 2.

**Fig. 15:**
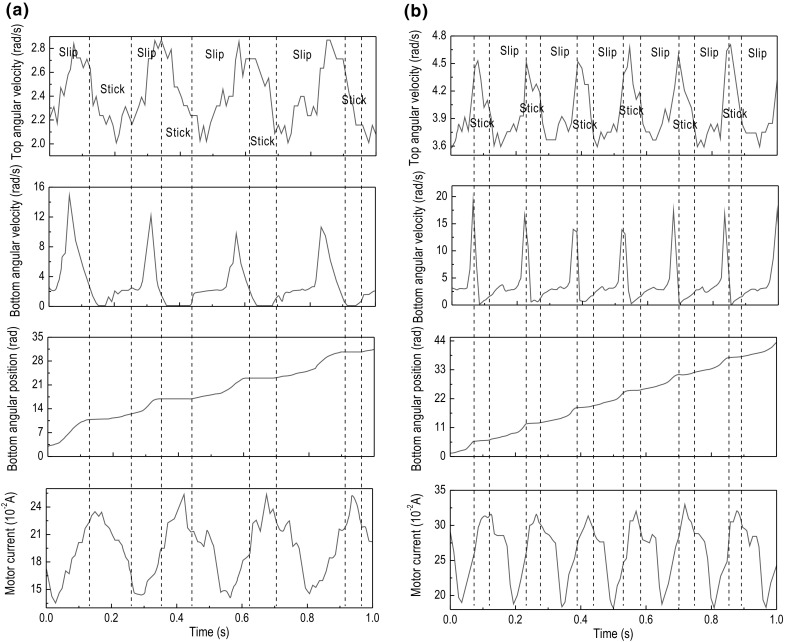
Experimental results of Test 6. The time histories of top angular velocity, bottom angular velocity, bottom angular position, and motor current are presented for $\kappa =2.46\times 10^{-2}\, \mathrm {m}^4$, $k=3699.73$ N/m, $\zeta =1.32\times 10^{-2}$ J s/rad, and $T_b=-9.23$ Nm obtained at the motor speed of **a** 2.7 rad/s and **b** 4.6 rad/s

To measure both restoring torque in the shafts $\tau$ and therefore the frictional torque $T_b$, the angle of twist $\theta$ has to be determined. Following the concept of a torque gauge tool, a $360^{\circ }$ protractor was added to the experimental rig. Since the drill-bit was contacted with the rock sample, when the motor began to supply torque *u*, the flexible shaft began to twist from one end. This meant that a restoring torque was building up in the flexible shaft, and a frictional torque was also being exhibited on the drill-bit. Eventually, when the restoring torque $\tau$ exceeded the frictional torque $T_b$, the drill-bit started to move. Before the slip occurred, there was a brief moment where the restoring torque in the shaft was equal to the friction torque, i.e., $\tau =T_b$. Therefore, by using the protractor, the angle of twist $\theta$ at the point in which the drill-bit started to move can be determined. This angle of twist $\theta$ was then used in the relationship $\tau =-\,\kappa \,\theta$ to calculate the restoring torque $\tau$, leading to the determination of the friction torque on the drill-bit, $T_b$. Finally, three levels of frictional torque $T_b$ have been obtained which are presented in Table 2.

## Experimental results

This section presents a number of different stick–slip scenarios obtained experimentally using two flexible shafts under variations in the system parameters, such as motor rotary speed and frictional torque, which in turn can be adjusted by changing the WOB. Table 3 presents the number of tests conducted along with the parameters of the drill-string during each test. As can be seen from the table, each setting of the drill-string was subjected by a low and high speed, denoted by (a) and (b) in Table 3, respectively. The experimental results obtained from the LabVIEW interface are displayed in Figs. 10, 11, 12, 13, 14 and 15, where the velocities at the top and bottom of the drill-string are presented in the two upper panels, and the displacement at the bottom of the drill-string and the current of the motor are given in the two lower panels.

The experimental tests revealed that for shaft 1, which has the lower spring constant and higher damping constant, the stick–slip phenomenon is clearly observable. When the drill-bit enters the stick phase, it becomes stationary for a brief period which is seen on the angular time graph of the bottom encoder, as at this instance, the velocity drops to 0 rad/s. However, the stick–slip phenomenon is not properly visible in the tests conducted with the second shaft, as seen in the results of the tests shown in Figs. 13, 14 and 15. In fact, the tests presented in Fig. 15 are the only tests with the second shaft showing the speed of the bottom encoder dropping to 0 rad/s, meaning that the drill-bit is in the stick phase. This is most likely due to the fact that the second shaft used in these tests is of a much higher spring constant than that of the first shaft. As the spring constant is increased, the net force in the spring becomes larger and the frictional force struggles to surpass it, leading to a reduction in the occurrence of stick–slip. Another factor is due to the damping constant in the second shaft, which is less than that in the first shaft.

It is also worth noting that the durations of the stick phase, in the cases where stick–slip occurs, are smaller in the tests done with shaft 2 than with shaft 1. This gives an indication that the spring constant of the shaft affects the period of the stick–slip oscillations, i.e., increasing spring constant decreases periods of stick, for the same levels of frictional torque. This is consistent from a physical point of view, because if the shaft is stiffer, it means that it will require a greater frictional torque, or reaction torque, in order to cause the drill-string to enter the stick phase or remain in the stick phase for a longer period of time. Nevertheless, this effect on the duration can also be caused by the difference in the damping constant, as the second shaft has a lower damping constant than the first.

In Fig. 10a, showing the results from test 1 (a) (see Table 3), stick–slip is observed when the rotary table speed was set to 2.7 rad/s and the frictional torque was set to -2.30 Nm. When comparing this test with the one shown in Fig. 10b, where the frictional torque is kept the same, but the rotary table speed is increased to 5.3 rad/s, a decrease in the period of the stick phase can be seen. This observation was also made when carrying out a comparison of Fig. 11a, b, as well as when comparing Fig. 12a, b, whereby in each set of tests, the only parameter to change was the speed of the rotary table and therefore speed of the drill-bit. Evidently, this is due to the fact that an increasing speed of the drill-bit accelerates the torsional oscillations, due to which the period of the stick–slip cycles decreases.

Continuing on from the discussion on the effects of rotary table speed on stick–slip, the frequency of stick–slip between the tests shown in Fig. 10a, b was seen to stay the same, but in tests 2 (a) and 2 (b), this frequency was seen to increase. This is because when the rotary speed from the motor was increased, the resulting decrease in the stick–slip period would allow for the drill-bit to enter the stick phase more often, for a given period of time. These observations are also made in Figs. 13, 14 and 15, where the flexible shaft was replaced with one of the higher spring constants.

The experimental measurements depicted in Figs. 10a, 11a and 12a show an increase in the occurrence of stick–slip, the period of stick–slip and the amplitude in the burst of speed exhibited immediately after the drill-bit exits the stick phase. In these tests, the only parameter to change was the frictional torque applied to the drill-bit; therefore, this suggests that an increase in the frictional torque applied to the drill-bit increases the three attributes of stick–slip mentioned. This observation was also made when comparing the results from tests shown in Figs. 10b, 11b and 12b, which is consistent with the argument outlined above.

In each of the tests conducted, the current levels of the motor were monitored. Through careful inspection of each of the results obtained, it can be seen that when the drill-bit enters the stick phase, there is a spike in the current. This is because when the frictional torque causes the drill-bit to become stationary or decrease significantly in speed, the load on the motor is increased, which in turn produces an increment in the current, as is typical for DC motors. This then causes the rotational speed of the motor to decrease slightly, due to the fact that the motor is relatively small. Once the drill-bit transitions from the stick phase to the slip phase, the current is seen to decrease by a certain margin. This again is because in DC motors the current through the motor is inversely proportional to the rotational speed; therefore, as the rotational speed increases once the drill-bit has transitioned to the slip phase, the current decreases.

## Conclusions

The main goal of this investigation was to carry out a numerical and an experimental parametric study on the stick–slip phenomenon observed in downhole drilling, using a small-scale drilling rig. The experimental tests were conducted based on the system design platform LabVIEW, which was used to control the rig operation, read the measurements from the encoders and process and visualize the collected data, such as angular velocity, angular displacement and motor current. The main parameters of the system were identified, which include the spring constants of the two flexible shafts used, as well as the damping constants and torsion constants.

In the first part of the paper (Sect. 2), we presented a preliminary numerical study of a lumped-parameter model of a drill-string, as shown in the schematic diagram given in Fig. 2. For this purpose, we employed path-following (continuation) techniques for non-smooth systems implemented via the continuation platform COCO [25, 26] (see also [32–34] for some recent applications of these techniques). This allowed studying the complex bifurcation scenario of the considered drill-string model, when considering the weight on bit (WOB) as the bifurcation parameter. The analysis revealed the important role played by the WOB in order to control the dynamical behavior of the system. The sequence of bifurcations detected during the continuation is shown in Fig. 4a. For low values of WOB, the system presents a stable equilibrium corresponding to constant rotation, as physically expected. Our study indicates, however, that this equilibrium loses stability if the WOB exceeds a critical value, defined by a subcritical Hopf bifurcation (H). This gives rise to a branch of unstable periodic solutions (with no stick–slip), which usually would remain undetected via direct numerical integration. The continuation approach, however, allowed us to trace this branch and discover that the unstable periodic solutions become stable stick–slip solutions via a grazing–sliding bifurcation (GR-SL). Furthermore, the numerical study showed that there is a parameter window defined by the H and GR-SL points in which the drill-string system is multistable. Specifically, in this window there exists three attractors: one corresponding to constant rotation, one to stick–slip oscillation, and one to permanent sticking, which occurs for large values of WOB, as physically expected. Therefore, in this parameter window the system presents multistability, a property that can be exploited to steer the response from, e.g., a sticking equilibrium or stick–slip oscillation to an equilibrium with constant drill-bit rotation.

The stick–slip behavior was further investigated using the experimental rig described in Sect. 3.1. From the results, it was established that the effects of the stick–slip phenomenon can be controlled by various parameters. These include the speed of the rotary table, the spring constant (or stiffness) of the flexible shaft used to resemble the drill-pipes, the torsion constant of the flexible shaft, the damping constant of the flexible shaft, and the frictional torque exhibited on the drill-bit, which in turn can be significantly affected by the WOB, as the predicted by the preliminary numerical study. Furthermore, it was established that increasing the spring constant reduced the period of the stick phase in stick–slip oscillations and ultimately reduced the occurrence of the whole phenomenon. Unfortunately, due to the slender nature and lengths of the drill-pipes used in downhole drilling, it is not the most viable of methods for suppressing stick–slip oscillations. Decreasing the damping constant was also seen to decrease the occurrence of stick–slip; however, the damping constants of the two shafts in this investigation were quite similar to each other. Therefore, further research in this direction will be required in order to gain a deeper insight into the effects of damping constants on stick–slip.

The main results concerning the experimental study presented here are related to the effects of frictional torque on the drill-bit, in regard to stick–slip, which are closely related to the WOB applied to the drill-string, as was shown by the preliminary numerical study presented in Sect. 2. It was found that increasing the frictional torque, i.e., the reaction torque, increased all major attributes of stick–slip, such as the period of stick phase and the amplitude in the burst of speed exhibited immediately after the drill-bit exits the stick phase. Since frictional torque on the drill-bit is a resultant of the WOB, adjusting the WOB can be used as an effective control strategy to suppress stick–slip oscillations; see, e.g., [12]. However, care must be taken as excess reduction in the WOB, and therefore, frictional torque could nullify the whole cutting process in the bit–rock interactions. Furthermore, our experimental investigation revealed that during the stick–slip oscillations, the motor experienced spikes in the current. This was found to occur because the load on the motor shaft was increased during the stick phase of the drill-bit and in DC motors, a decrease in the rotational speed results in an increase in current.

Future work will include enhancements of the proposed experimental rig in terms of its dimensions, hence allowing the use of multiple flexible shafts connected in series, with the purpose of simulating the varying length of drill-strings in real applications (see Fig. 2b). In connection to this, the augmented experimental rig can be used to calibrate the drill-string model studied in Sect. 2, in such a way to achieve a sound agreement between theoretical and experimental observations. This will allow a series of experimental and numerical tests of different control mechanisms to suppress stick–slip oscillations, based on previous theoretical investigations (see, e.g., [3]).

## References

[1] Kamel JM, Yigit AS (2014). Modeling and analysis of stick–slip and bit bounce in oil well drillstrings equipped with drag bits. J. Sound Vib..

[2] Kapitaniak M, Vaziri Hamaneh V, Páez Chávez J, Nandakumar K, Wiercigroch M (2015). Unveiling complexity of drill–string vibrations: experiments and modelling. Int. J. Mech. Sci..

[3] Liu Y (2015). Suppressing stick–slip oscillations in underactuated multibody drill-strings with parametric uncertainties using sliding-mode control. IET Control Theory Appl..

[4] Ghasemloonia A, Rideout DG, Butt SD (2015). A review of drillstring vibration modeling and suppression methods. J. Pet. Sci. Eng..

[5] Richard T, Germay C, Detournay E (2007). A simplified model to explore the root cause of stick–slip vibrations in drilling systems with drag bits. J. Sound Vib..

[6] Germay C, de Wouw NV, Nijmeijer H, Sepulchre R (2009). Nonlinear drillstring dynamics analysis. SIAM J. Appl. Dyn. Syst..

[7] Liao, C.M., Balachandran, B., Karkoub, M., Abdel-Magid, Y.L.: Drill-string dynamics: reduced-order models and experimental studies. J. Vib. Acoust. **133**, 041008:1-8 (2011)

[8] Liu X, Vlajic N, Long X, Meng G, Balachandran B (2013). Nonlinear motions of a flexible rotor with a drill bit: stick–slip and delay effects. Nonlinear Dyn..

[9] Nandakumar K, Wiercigroch M (2013). Stability analysis of a state dependent delayed, coupled two DOF model of drill-string vibration. J. Sound Vib..

[10] Navarro-López EM, Cortés D (2007). Avoiding harmful oscillations in a drillstring through dynamical analysis. J. Sound Vib..

[11] Puebla H, Alvarez-Ramirez J (2008). Suppression of stick–slip in drillstrings: a control approach based on modeling error compensation. J. Sound Vibr..

[12] Canudas-de-Wit C, Rubio FR, Corchero MA (2008). D-OSKIL: a new mechanism for controlling stick–slip oscillations in oil well drillstrings. IEEE. Trans. Control Syst. Technol..

[13] Leonov GA, Kuznetsov NV, Kiseleva MA, Solovyeva EP, Zaretskiy AM (2014). Hidden oscillations in mathematical model of drilling system actuated by induction motor with a wound rotor. Nonlinear Dyn..

[14] Liu Y, Yu HN (2013). A survey of underactuated mechanical systems. IET Control Theory Appl..

[15] Liu, Y.: Control of a class of multibody underactuated mechanical systems with discontinuous friction using sliding-mode. Trans. Inst. Meas. Control (2016). 10.1177/0142331216661759

[16] Melakhessou H, Berlioz A, Ferraris G (2003). A nonlinear well-drillstring interaction model. J. Vib. Acoust..

[17] Mihajlovic N, van de Wouw N, Hendriks MP, Nijmeijer H (2006). Friction induces limit cycling in flexible rotor systems: an experimental drillstring setup. Nonlinear Dyn..

[18] Khulief YA, AI-Sulaiman FA (2009). Laboratory investigation of drillstring vibration. J. Mech. Eng. Sci. Part C.

[19] Lu, H., Dumon, J., de Wit, J.: Experimental study of the D-OSKIL mechanism for controlling the stick–slip oscillations in a drilling laboratory testbed. In: Proceedings of the Multi-Conference on Systems and Control, (Saint Petersburg, Russia), pp. 1551–1556 (2009)

[20] Forster, I., Macfarlane, A., Dinnie, R.: Asymmetric vibration damping tool—small scale rig testing and full scale field testing. In: Proceedings of the SPE/IADC Drilling Conferenc and Exhibition, (New Orleans Louisiana, USA), p. 128459 (2010)

[21] Patil PA, Teodoriu C (2013). A comparative review of modelling and controlling torsional vibrations and experimentation using laboratory setups. J. Pet. Sci. Eng..

[22] Navarro-López EM, Cortés D (2007). Avoiding harmful oscillations in a drillstring through dynamical analysis. J. Sound Vib..

[23] Navarro-López EM, Licéaga-Castro E (2009). Non-desired transitions and sliding-mode control of a multi-DOF mechanical system with stick-slip oscillations. Chaos Solitons Fractals.

[24] Navarro-López EM (2009). An alternative characterization of bit-sticking phenomena in a multi-degree-of-freedom controlled drillstring. Nonlinear Anal. Real World Appl..

[25] Dankowicz H, Schilder F (2013). Recipes for Continuation.

[26] Dankowicz H, Schilder F (2011). An extended continuation problem for bifurcation analysis in the presence of constraints. J. Comput. Nonlinear Dyn..

[27] Awrejcewicz J, Olejnik P (2005). Friction pair modeling by a 2-DOF system: numerical and experimental investigations. Int. J. Bifurc. Chaos.

[28] Kuznetsov YA (2004). Elements of Applied Bifurcation Theory, vol. 112 of Applied Mathematical Sciences.

[29] di Bernardo M, Budd CJ, Champneys AR, Kowalczyk P (2004). Piecewise-smooth dynamical systems. Theory and Applications, vol. 163 of Applied Mathematical Sciences.

[30] Mohazzabi P, Shefchik BM (2001). A universal relationship between spring constant and torsion constant. J. Phys. Chem. Solids.

[31] Yamada Y (2007). Materials for Springs.

[32] Liao M, Ing J, Páez Chávez J, Wiercigroch M (2016). Bifurcation techniques for stiffness identification of an impact oscillator. Commun. Nonlinear Sci. Numer. Simul..

[33] Páez Chávez J, Voigt A, Schreiter J, Marschner U, Siegmund S, Richter A (2016). A new self-excited chemo-fluidic oscillator based on stimuli-responsive hydrogels: mathematical modeling and dynamic behavior. Appl. Math. Model..

[34] Liu Y, Páez Chávez J (2017). Controlling multistability in a vibro-impact capsule system. Nonlinear Dyn..

